# Differentiating amyloid beta spread in autosomal dominant and sporadic Alzheimer’s disease

**DOI:** 10.1093/braincomms/fcac085

**Published:** 2022-04-13

**Authors:** Elizabeth Levitis, Jacob W Vogel, Thomas Funck, Vladimir Hachinski, Serge Gauthier, Jonathan Vöglein, Johannes Levin, Brian A Gordon, Tammie Benzinger, Yasser Iturria-Medina, Alan C Evans, for the Dominantly Inherited Alzheimer Network, for the Alzheimer’s Disease Neuroimaging Initiative

**Affiliations:** 1 Montreal Neurological Institute, McGill University, Montreal, QC, Canada; 2 Western University, London, Ontario, Canada; 3 McGill Centre for Studies in Aging, McGill University, Montreal, QC, Canada; 4 German Center for Neurodegenerative Diseases, Munich, Germany; 5 Department of Neurology, Ludwig-Maximilians-Universität München, Munich, Germany; 6 Munich Cluster for Systems Neurology (SyNergy), Munich, Germany; 7 Department of Radiology, Washington University School of Medicine in Saint Louis, St Louis, Missouri, USA

**Keywords:** amyloid beta, Alzheimer’s disease, diffusion models, brain networks

## Abstract

Amyloid-beta deposition is one of the hallmark pathologies in both sporadic Alzheimer’s disease and autosomal-dominant Alzheimer’s disease, the latter of which is caused by mutations in genes involved in amyloid-beta processing. Despite amyloid-beta deposition being a centrepiece to both sporadic Alzheimer’s disease and autosomal-dominant Alzheimer’s disease, some differences between these Alzheimer’s disease subtypes have been observed with respect to the spatial pattern of amyloid-beta. Previous work has shown that the spatial pattern of amyloid-beta in individuals spanning the sporadic Alzheimer’s disease spectrum can be reproduced with high accuracy using an epidemic spreading model which simulates the diffusion of amyloid-beta across neuronal connections and is constrained by individual rates of amyloid-beta production and clearance. However, it has not been investigated whether amyloid-beta deposition in the rarer autosomal-dominant Alzheimer’s disease can be modelled in the same way, and if so, how congruent the spreading patterns of amyloid-beta across sporadic Alzheimer’s disease and autosomal-dominant Alzheimer’s disease are. We leverage the epidemic spreading model as a data-driven approach to probe individual-level variation in the spreading patterns of amyloid-beta across three different large-scale imaging datasets (2 sporadic Alzheimer’s disease, 1 autosomal-dominant Alzheimer’s disease). We applied the epidemic spreading model separately to the Alzheimer’s Disease Neuroimaging initiative (*n* = 737), the Open Access Series of Imaging Studies (*n* = 510) and the Dominantly Inherited Alzheimer’s Network (*n* = 249), the latter two of which were processed using an identical pipeline. We assessed inter- and intra-individual model performance in each dataset separately and further identified the most likely subject-specific epicentre of amyloid-beta spread. Using epicentres defined in previous work in sporadic Alzheimer’s disease, the epidemic spreading model provided moderate prediction of the regional pattern of amyloid-beta deposition across all three datasets. We further find that, whilst the most likely epicentre for most amyloid-beta–positive subjects overlaps with the default mode network, 13% of autosomal-dominant Alzheimer’s disease individuals were best characterized by a striatal origin of amyloid-beta spread. These subjects were also distinguished by being younger than autosomal-dominant Alzheimer’s disease subjects with a default mode network amyloid-beta origin, despite having a similar estimated age of symptom onset. Together, our results suggest that most autosomal-dominant Alzheimer’s disease patients express amyloid-beta spreading patterns similar to those of sporadic Alzheimer’s disease, but that there may be a subset of autosomal-dominant Alzheimer’s disease patients with a separate, striatal phenotype.

## Teaser

Using an epidemic spreading model and three independent datasets, Levitis *et al.* show evidence for transneuronal amyloid spread in sporadic and autosomal-dominant Alzheimer’s disease. Whilst most autosomal dominant patients showed a similar spread pattern to sporadic Alzheimer’s disease, a separate phenotype with younger onset and a striatal phenotype emerged.

## Graphical Abstract

**Graphical Abstract:**
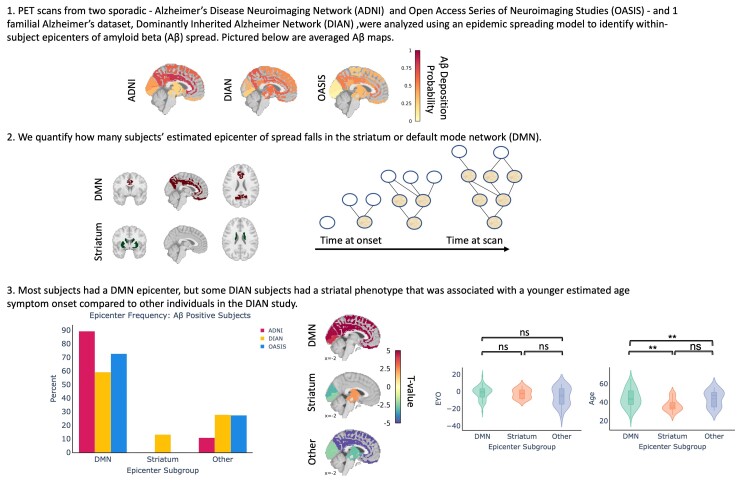

## Introduction

To date, there is no cure for Alzheimer’s disease (AD), the principal neurodegenerative cause of dementia. Treating patients with dementia is costly—in 2009, the average cost for a patient with AD was roughly 57 000 USD.^1^ The socioeconomic gravity of treating AD has spurred research seeking to prevent or mitigate AD by first developing biomarkers that can be used for early diagnosis and monitoring.^2^ The two main pathological signs of AD are neurofibrillary tau tangles and amyloid-beta senile plaques, and both are required to definitively confirm AD at autopsy.^3^ Most hypothetical models of AD progression have been rooted in the amyloid cascade hypothesis, which posits that excessive amounts of soluble amyloid-beta cause a buildup of insoluble amyloid-beta, disrupting synaptic function and accelerating tau hyperphosphorylation.^4^ The majority of AD cases are sporadic in nature, and the much rarer autosomal dominant form of AD is caused by mutations in genes—namely, *APP*, *PSEN1*, *PSEN2*—that impact the processing of the amyloid precursor protein from which the amyloid-beta peptide is cleaved. Whilst ample research has pointed to accumulation of amyloid-beta in the brain as being one of the earliest pathological biomarkers in both sporadic Alzheimer’s disease (sAD) and autosomal-dominant Alzheimer’s disease (ADAD), we know quite little about where and how amyloid-beta begins to accumulate, how it spreads in the brain and whether either of these is variable across individuals.

amyloid-beta is purported to spread trans-neuronally—along neuronal connections—in a prion-like fashion as opposed to spreading locally in the extracellular space.^5^ Earlier evidence of this mechanism came from the animal model literature where infused amyloid-beta was shown to travel between neurones across neuronal fibres.^6^ Computational modelling quantitatively comparing the two modes of spread has lent further support to the trans-neuronal spread as the more likely mechanism via which amyloid-beta propagates throughout the brain.^7^ An ADAD mutation virtually guarantees amyloidosis, making carriers of these mutations incredibly important for the study of amyloid-related processes and brain changes in AD. However, it is still unclear just how similar ADAD and sAD are with respect to the progression of various biomarkers, including amyloid-beta.

In general, most studies in this domain have focused less on inter-individual variability and have primarily reported group differences. Unlike in sAD, where amyloid-beta deposition is highest in neocortical areas, several groups have reported significantly increased striatal, thalamic and neocortical amyloid-beta deposition in ADAD mutation carriers compared with noncarriers.^8,9^ One study evaluating differences between the presenilin 1 (PSEN1), presenilin 2 (PSEN2) and amyloid precursor protein (APP) ADAD mutation types found that all mutation types had high striatal PiB binding, whilst some mutation carriers had higher cortical PiB binding. Interestingly, PiB binding in the cortex was found to be lower in ADAD mutation carriers than age-matched subjects with probable sAD.^10^ Whilst the sample size of this study was small (*n* = 30 ADAD mutation carriers, *n* = 30 sAD subjects), the findings suggest that the most probable area(s) of earliest amyloid-beta accumulation may not be homogenous amongst all ADAD mutation carriers.

Recently, an event-based model of disease progression was applied to ADAD mutation carriers. The authors found that the biomarker likeliest to exhibit the earliest deviation from normal levels was a cortical amyloid-beta deposition measure, followed by amyloid-beta deposition in the caudate, putamen, accumbens and thalamus.^11^ In sAD, a separate model leveraged cerebrospinal fluid (CSF) and amyloid-beta signals to stage subjects according to amyloid-beta accumulation status. In this study, subjects who were both CSF and amyloid-beta negative according to a set of data-driven thresholds were deemed to be non-accumulators, whereas those who were CSF positive but amyloid-beta negative were deemed to be early amyloid-beta accumulators.^12^ Regions pinpointed as areas of earliest accumulation were those that had significantly increased amyloid-beta signal in early accumulators compared with non-accumulators. According to this system, the precuneus, medial orbitofrontal cortex and posterior cingulate (PC) were all categorized as regions of early accumulation, whereas the caudal anterior cingulate (CAC) was pinpointed as an area of intermediate accumulation. Together, these studies suggest possible differences between ADAD and sAD in the earliest regions to accumulate amyloid-beta.

Whilst both data-driven approaches can be used to glean the order in which biomarkers can be detected at either a regional or global level, neither of them is mechanistic in nature. To better understand how amyloid-beta or tau spreads in the brain, we can instead turn to an epidemic spreading model (ESM) developed to stochastically reproduce the propagation and deposition of misfolded proteins such as amyloid-beta, tau and alpha-synuclein. The overarching nonlinear differential equation of the model posits that the change in misfolded protein deposition in each macroscopic region of interest (ROI) is equal to the probability of endogenously producing and exogenously receiving misfolded proteins from connected ROIs, minus the probability of clearing the deposited misfolded proteins. This approach has previously been applied to model the spread of amyloid-beta and tau across anatomical connections in individuals along the sAD spectrum.^13,14^ When applied to over 700 subjects in the ADNI dataset, the ESM was able to explain 46–57% of the variance in the mean regional amyloid-beta deposition probabilities of the distinct clinical subgroups and identified the posterior and anterior cingulate cortices as the seed regions of amyloid-beta propagation. These seed regions are in agreement with what has been established in the literature.^15^ Using the ESM, we can evaluate whether there is sufficient evidence to suggest that amyloid-beta spreads along neuronal connections in ADAD as well. Furthermore, we can evaluate how similar ADAD and sAD are with respect to which regions amyloid-beta begins spreading from. We tackle this question by applying the ESM within three different datasets representing sAD and ADAD, to both evaluate differences between ADAD and sAD, as well as validate the previously published results in an independent dataset.

## Methods

### Participants

Participants for this study are comprised of individuals from three multi-centre studies: the Dominantly Inherited Alzheimer Network (DIAN; https://dian.wustl.edu), the Alzheimer’s Disease Neuroimaging Initiative (ADNI; http://adni.loni.usc.edu) and the Open Access Series of Imaging Studies (OASIS; www.oasis-brains.org). Whilst the ESM had already been applied to ADNI, a dataset representative of LOAD, we include an additional dataset for two reasons—(i) to validate the previously published results in an independent cohort and (ii) to compare results in DIAN with a dataset that used the same radiotracer and was processed using the same pipeline.

The DIAN dataset represents individuals from families known to have mutations in the *APP*, *PSEN1* and *PSEN2* genes. Both mutation carriers and non-carriers were used for different stages of the analysis. We selected individuals who had at least one PIB positron emission tomography (PET) scan and accompanying T1w scan from the 12th semiannual DIAN data freeze. For this study, DIAN serves as the dataset representative of ADAD.

The OASIS dataset is a compilation of participants from multiple studies, and the participants range from older, cognitively normal adults to those at various stages of cognitive decline and dementia.

### MRI and PET acquisition and preprocessing

MRI and PET acquisition procedures for the DIAN,^16^ ADNI (http://adni.loni.usc.edu/methods/), and OASIS^17^ datasets have previously been described in detail.

It is important to note that the processing pipeline and the radiotracer for the ADNI dataset diverge from those used for DIAN and OASIS. One of the overarching goals of this study was to test model robustness both across datasets and across methodologies. The multi-cohort design of our study allows us to (i) compare ESM performance in ADAD (DIAN) to sAD (OASIS) where both datasets were acquired using the same radiotracer and processed using the same pipeline and (ii) ascertain whether the results reported in the original ESM publication for ADNI could be replicated in a second sAD dataset using a different pipeline.

For ADNI, the preprocessing pipeline is taken from the original ESM publication.^13^ Briefly, individual AV45 PET scans were acquired and processed in the following order—dynamic co-registration, averaging across time, re-sampling and reorientation from native space to a standard voxel space, spatial filtering and finally spatial normalization to MNI space. For the DIAN and OASIS dataset, whole-brain T1w scans and individual PiB-PET scans were acquired. Quality control was performed as per the ADNI protocol. FreeSurfer version 5.3 (http://surfer.nmr.mgh.harvard.edu) was used to derive subject-specific segmentations corresponding to regions in the Desikan–Killiany–Tourville atlas (DKT).^18^ Only cortical and subcortical regions overlapping with the Mindboggle DKT atlas were used, for a total of 78 regions.^19^

For both OASIS and DIAN, the PET Unified Pipeline (PUP; https://github.com/ysu001/PUP) was used to preprocess the PET scans. The processing steps used include smoothing, interframe motion correction and co-registration. Specifically, PET images in the 4dfp format are smoothed to achieve a common spatial resolution of 8 mm to minimize inter-scanner differences.^20^ PET-MR registration was performed using a vector-gradient algorithm (VGM).^21^ Co-registered summed PET scans in the 4dfp file format were downloaded from the CNDA portal (https://cnda.wustl.edu), and 4dfp images were subsequently converted to the Nifti file format for further analysis.

### Regional amyloid-beta probabilities

Traditionally, static PET processing involves quantifying co-registered PET images using standardized uptake value ratios (SUVR) for each ROI with respect to the average signal in a reference region devoid of specific tracer binding. The reference region typically used in AD amyloid-beta PET imaging studies is the cerebellar cortex; however, amyloid deposition has been observed in the cerebellar cortex of individuals with ADAD.^22^ Based on recent work seeking to clarify the optimal reference region for amyloid-beta measurement using PiB-PET and the DIAN cohort, we used the brainstem as the reference region for the DIAN and OASIS datasets.^23,24^ For the ADNI dataset, we used the amyloid-beta deposition probabilities that had previously been generated (using a cerebellar reference region).^13^

The original ESM paper introduced a voxelwise probability metric to calculate amyloid-beta deposition probabilities. For each subject, this approach creates a bootstrapped sampling consisting of 40 000 subsamples in the 5–95% of values in the reference region. Subsequently, an extreme value distribution (EVD) is created using the maximum value observed in each bootstrapped sample. The EVD is used to create an extreme cumulative distribution function, and for each voxel in the PET image, the probability of it being greater than every value in the EVD is computed. A final regional amyloid-beta deposition probability is calculated as the average of the probabilities corresponding to each voxel within a given ROI. Given the overall higher PiB-PET signal in the brainstem than the cerebellar cortex, we use the 75th percentile value rather than the maximum in each bootstrap sampling to create the EVD when using the brainstem as the reference region.

For the DIAN dataset, we observed that noncarriers’ amyloid-beta deposition probabilities were negligible in all ROIs except for the globus pallidus and thalamus, ROIs that have previously been observed to have nonspecific uptake of PiB.^25,26^ Given their young age (Table 1), we are confident that the DIAN non carriers are truly amyloid-negative and are therefore a fully reliable control group. Subsequently, for each ROI, across all available timepoints, the noncarriers’ signal was used to create a ROI-specific control distribution. For each mutation carrier, we calculated a *Z*-score for their amyloid-beta binding probability in the ROI with respect to the ROI-specific control distribution. Within each ROI, we min–max scaled the absolute values of the z-scored signal across all timepoints to have probabilities in the [0,1] range again.

**Table 1:** Demographic information

Dataset	DIAN		ADNI	OASIS
	T1	T2		
*n*	249	124	737	510
Age (SD)	39.01 (10.7)	42.12 (9.7)	72.43 (7.2)	67.65 (9.8)
% Women	56.3%	60.1%	44.9%	57.8%
EYO (SD)	−8.54 (10.9)	−4.7 (9.8)	NA	NA
% ApoE4	30.1%	29.53%	51.7%	NA
% amyloid-beta positive	55%	63.7%	54%	25%
% Cognitively normal	68.7%	58.8%	26.2%	86.5%

EYO, estimated years to symptom onset; T1, timepoint 1; T2, timepoint 2; NA, not applicable.

### Epidemic spreading model

The spread of amyloid-beta was simulated using the ESM, a diffusion model that has previously been used to simulate the spread of amyloid-beta and tau in the ADNI dataset from an initial epicentre(s) and through an ROI network.^13,14^ In addition to the connectivity between ROIs, subject-specific propagation parameters influence the magnitude or extent of the spreading pattern. These parameters correspond to a global clearance rate, global production rate and age of onset. These are fit by solving a non-linear differential equation designed to reproduce the overall regional pattern of amyloid-beta deposition. The ESM is fit by searching the parameter space, and the set of parameters that yield the regional pattern of amyloid-beta deposition most like the reference (observed) pattern is selected.

The main data input to the ESM is the ROI by Subject matrix reflecting regional amyloid-beta deposition probabilities for each subject. Epicentres can either be supplied by the user or selected in a data-driven way. In the data-driven context, the ROI or combination of ROIs that best explain the average group-level pattern are returned as the epicentres. For a more detailed overview of the equations underlying the ESM, please refer to the original publication.^13^

### Connectivity measures

In order to propagate amyloid-beta signal across the brain, the ESM requires a matrix of pairwise relationships between ROIs. This informs the final regional pattern of amyloid-beta. Earlier applications of the ESM tested whether amyloid-beta spreads along synapses by using a structural connectivity matrix.

We used a structural connectivity matrix derived from diffusion spectrum imaging (DSI) scans of 60 young healthy subjects from the CMU-60 DSI template.^27^ The acquisition and pre-processing steps have been described in detail in the original ESM paper and were based on methodology developed in an earlier paper.^28^ Briefly, all images were non-linearly co-registered to MNI space, and orientation distribution functions (ODFs) representing nerve fibre orientations were calculated. All intravoxel fibre ODF maps were averaged to create an ODF template, and an automated fibre tractography method was used to calculate probabilistic axonal connectivity values for each voxel and the surface of each grey matter region in the DKT atlas. Previously described anatomical connection probabilities were then generated for each ROI–ROI pair.

### amyloid-beta positivity

The ESM has previously been shown to be sensitive to spurious levels of signal, so we opted to confine our analysis to amyloid-beta positive subjects.^14^ We used Gaussian mixture modelling (GMM) to compute amyloid-beta positivity thresholds in a data-driven way for both the PUP generated SUVRs and the probability values averaged across a composite set of regions that are implicated in AD. Specifically, these include the bilateral precuneus, superior frontal, rostral middle frontal, lateral orbitofrontal, medial orbitofrontal, superior temporal and middle temporal ROIs. For each metric, we fit a two-component mixture model across the entire DIAN dataset—including non-carriers and mutation carriers—and estimated a cut-off. Only subjects who were positive on both the SUVR and probability metrics were considered amyloid-beta positive for subsequent analyses. Since the DIAN and OASIS datasets were both processed using the WUSTL PUP, we applied the cut-offs generated using the DIAN dataset to the OASIS dataset as well. amyloid-beta positive subjects in these datasets were defined as those whose average amyloid-beta value across a set of previously defined cortical areas surpassed 0.81 and 0.01236 for SUVR and deposition probability values, respectively. We illustrate the correspondence between within-subject composite amyloid-beta SUVRs and deposition probabilities, as well as the GMM results in Supplementary Fig. 1. amyloid-beta positive ADNI subjects were identified using a previously defined composite amyloid-beta SUVR threshold of 1.11.

### Statistical analysis

Using the structural connectivity matrix and the cross-sectional baseline subject by region amyloid-beta probability deposition matrix, we fit the ESM across different possible epicentres for the DIAN, ADNI, and OASIS datasets. Model performance for each experiment was evaluated by mean within-subject and global fit. Within-subject performance is evaluated as the Pearson *r*^2^ between the subject-specific observed and predicted regional amyloid-beta deposition probabilities measured using PiB-PET. We evaluate global fit by averaging the observed and predicted regional amyloid-beta probabilities across all subjects, respectively, and calculating the Pearson *r*^2^ between the averaged observed and predicted patterns. To ensure that our results are statistically significant and specific to the connectivity matrix we used, we scrambled the original connectivity matrix 100 times whilst preserving degree and strength distributions using the Brain Connectivity Toolbox (https://sites.google.com/site/bctnet/). We used the null distribution of the mean within-subject fit and global fit to calculate the mean and 95% confidence intervals (CIs) for each ESM experiment.

Building off previous results suggesting that amyloid-beta first accumulates in the PC and CAC and subsequently spreads to other regions in the brain (in ADNI), we sought to evaluate whether this finding replicates in another LOAD dataset, as well as the DIAN dataset. Given our objective of evaluating whether a cortical or striatal epicentre better explains amyloid-beta spreading patterns in ADAD, we repeated this analysis for all three datasets using the caudate and putamen as the seed regions.

For a more data-driven approach to epicentre selection, in each dataset, we evaluated global fit using each bilateral ROI as an independent epicentre. For each subject we noted the epicentre that provided the best within-subject fit, and we assessed how frequently specific epicentres were present within each dataset. Given the lack of consensus about whether ADAD mutation carriers first accumulate amyloid-beta in the striatum or neocortical regions that overlap with the default mode network, we further divided the possible epicentres into three subgroups—default mode network (DMN), striatum, and other. ROIs falling into the DMN group included the PC, cAC, rostral anterior cingulate, precuneus, and medial orbitofrontal cortex. The striatum subgroup included the caudate and putamen, and the other group contained all ROIs not in the other two subgroups. Using these data-driven epicentre subgroups, we compared within-subject model performance using either the caudate and putamen or CAC + PC as epicentres across the epicentre subgroups. We evaluated the statistical difference in the models’ performance across epicentre subgroups using the non-parametric Kolmogorov-Smirnov (K-S) test statistic.

After stratifying subjects across epicentre subgroups (DMN, Striatum and Other), we examined associations with age and EYO. We additionally ran ordinary least-squares general linear models (GLMs) to assess the relationship between the epicentre subgroup and the amyloid-beta signal in all ROIs whilst covarying for age and sex. We FDR corrected the relationships using the Benjamini–Hochberg approach. We additionally ran a multinomial logistic regression model to compare the ‘Other’, ‘DMN’ and ‘Striatum’ epicentre subgroups within the amyloid positive DIAN sample, given that the other datasets did not have any individuals who fell into the ‘Striatum’ epicentre sub-group. The covariates used were sex, CDR, age, and education.

As a follow-up, we evaluated the test-retest reliability of the best within-subject epicentre for each subject that had two scans in the DIAN dataset.

### Data availability

OASIS-3 and ADNI are open access datasets for which access can be obtained at https://www.oasis-brains.org/ and http://adni.loni.usc.edu/data-samples/access-data/, respectively. The DIAN data can be obtained by request through application, and more information about requesting data access can be found here https://dian.wustl.edu/our-research/for-investigators/dian-observational-study-investigator-resources/data-request-terms-and-instructions/.

### Code availability

The Matlab code for the ESM has been made available as a public software release with an accompanying paper (neuropm-lab.com/software^29^). All the Python code used to analyze ESM results, perform statistical analysis, and visualize results can be found at https://github.com/llevitis/DIAN_ESM_AmyloidBeta_Project.git.

## Results

### Sample information

Baseline PiB-PET scans measuring fibrillar amyloid-beta load were available for 249 ADAD mutation carriers in the DIAN dataset. One-hundred twenty-four of these mutation carriers had one follow-up PiB-PET scan, and 44 of them had two follow-up scans. Baseline AV45-PET scans were available for 737 individuals from the ADNI dataset, and baseline PiB-PET scans were available for 510 individuals from the OASIS cohort. Demographic information for these samples can be found in Table 1.

### Putative areas of early amyloid-beta accumulation in LOAD do not explain the full picture in ADAD

To evaluate whether neuronal connectivity can explain the whole-brain pattern of amyloid-beta in both ADAD mutation carriers and individuals from the OASIS dataset, we fit the ESM to regional amyloid-beta deposition probabilities derived using PiB-PET or AV45-PET data (see ‘Methods’ section).

**Figure 1:**
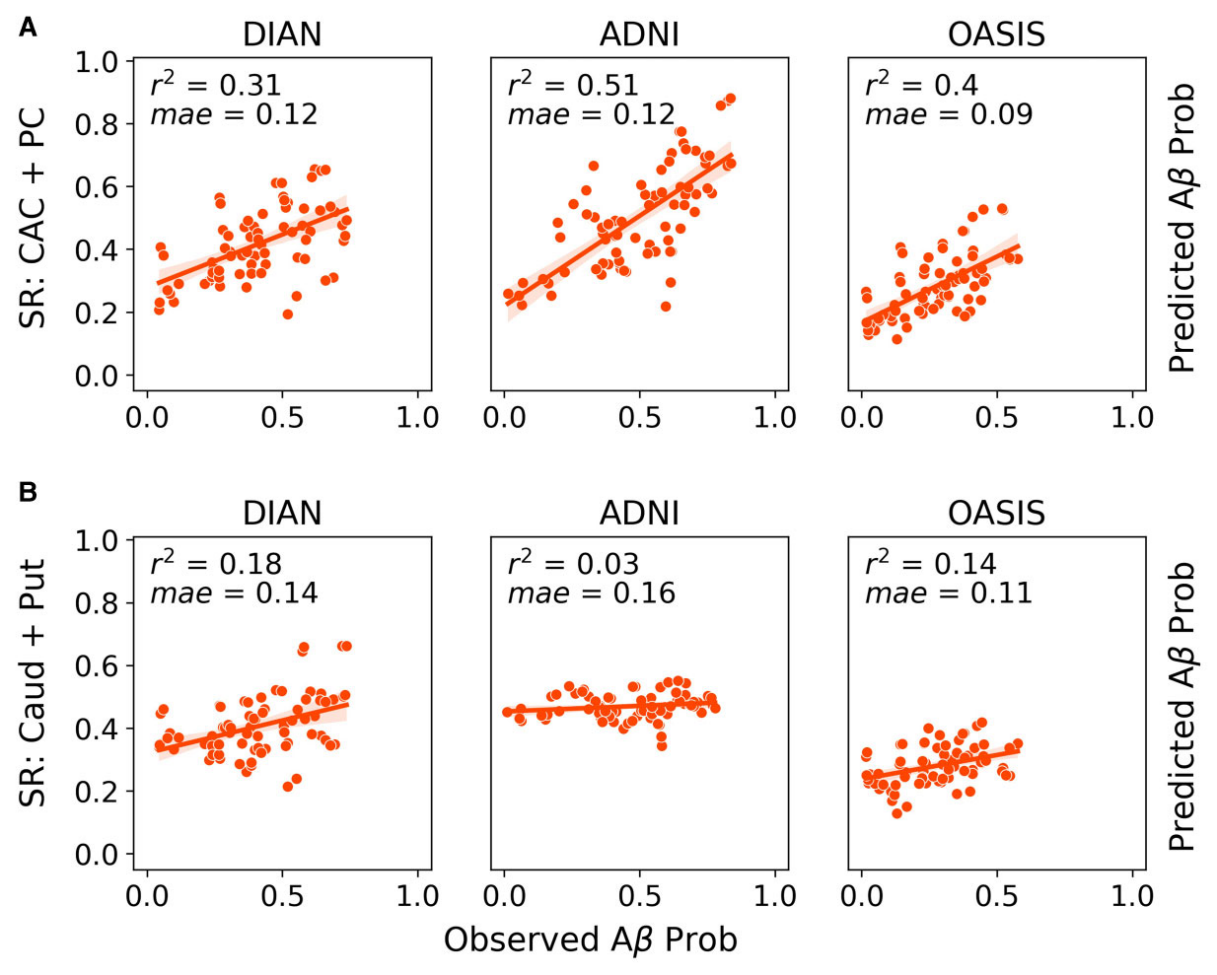
**Comparison of global model fit across datasets and epicentres.** ESM performance (global fit) across the ADNI, OASIS and DIAN datasets using either the (**A**) PC and caudal anterior cingulate or (**B**) caudate and putamen as epicentres. Each dot represents the observed and predicted mean signal for an ROI across all subjects within a dataset. Only amyloid-beta positive subjects were included.

We first evaluated how well previously identified regions of early amyloid, namely cingulate and striatal regions, recapitulate group-level whole-brain amyloid-beta patterns across all three datasets. We will refer to the model using the CAC and PC as epicentres as the CAC + PC model, and the one using the caudate and putamen as the striatal model. In the DIAN dataset, the model using the CAC and PC as seed regions explained 27% (null model mean *r*^2^ [95% CI] = 0.119 [0.089, 0.164]; *P* < 0.01) of the aggregated pattern of amyloid-beta, and on average explained 14.6% (null model mean *r*^2^ [95% CI] = 0.07 [0.002, 0.179]; *P* = 0.1) of the regional pattern of amyloid-beta within individual subjects (Fig 1A). In amyloid-beta positive subjects, the global fit and the mean within subject fit improved to 31% and 20.7% (*P* < 0.05), respectively. When stratifying performance across the three main mutation types, we found that there was no significant difference between the three groups.

In line with the results that had been previously shown for the ADNI dataset in,^13^ the CAC + PC model explained 53.9% (null model mean *r*^2^ [95% CI] = 0.103 [0.074, 0.148]; *P* < 0.01) of the aggregated pattern of amyloid-beta and on average explained 39.1% (null model mean *r*^2^ [95% CI] = 0.087 [0.002, 0.217]; *P* < 0.01) of the regional pattern of amyloid-beta in individual subjects. In amyloid-beta positive subjects, the global fit and the mean within subject fit changed slightly to 51% and 38%, respectively.

In the LOAD validation dataset, OASIS, the performance was lower than what had previously been reported for ADNI. Across the whole dataset, the CAC + PC model explained 28% (null model mean *r*^2^ [95% CI] = 0.158 [0.123, 0.217]; *P* < 0.01) of the aggregated pattern of amyloid-beta and on average explained 9% (null model mean *r*^2^ [95% CI] = 0.063 [0.017, 0.139]; *P* = 0.15) of the within subject variance. However, when we only look at amyloid-beta positive individuals, the global fit and the average within subject fit increased to 40% (null model mean *r*^2^ [95% CI] = 0.14 [0.098,0.18]; *P* < 0.01) and 21% (null model mean *r*^2^ [95% CI] = 0.082 [0.002, 0.196]; *P* = 0.04), respectively, and the results were significant.

Since a primary goal of this study was to identify whether a cortical or striatal epicentre better explains the regional patterns of amyloid-beta in DIAN, we additionally repeated the same analysis using the caudate and putamen as the seed regions (Fig. 1B). When applied to ADNI, the striatal model performed poorly. It explained 3% (null model mean *r*^2^ [95% CI] = 0.055 [0.043, 0.072]; *P* = 1) of the aggregated pattern of amyloid-beta and on average explained 5% (null model mean *r*^2^ [95% CI] = 0.05 [0.001, 0.139]; *P* = 0.5) of the within-subject amyloid-beta patterns in amyloid-beta positive subjects. In DIAN amyloid-beta positive subjects, the striatal model explained 18% (null model mean *r*^2^ [95% CI] = 0.103 [0.072, 0.146]; *P* < 0.02) of the aggregated pattern of amyloid-beta and on average explained 17.2% (null model mean *r*^2^ [95% CI] = 0.085 [0.001, 0.256]; *P* = 0.04) of the within-subject pattern. In amyloid-beta+ OASIS subjects, the striatal model explained 14% (null model mean *r*^2^ [95% CI] =0.084 [0.062, 0.133]; *P* < 0.02) and on average 11.4% (null model mean *r*^2^ [95% CI] = 0.059 [0.001, 0.16]; *P* = 0.15) of the global and within-subject results, respectively.

### Epicentre heterogeneity in DIAN compared with ADNI and OASIS

We initially compared ESM performance between the ADNI and DIAN dataset using a priori defined epicentres. Next, we ran the ESM using each bilateral ROI as the model epicentre to evaluate which ROI best explains the whole-brain patterns of amyloid-beta in each dataset. We assigned each participant to an epicentre subgroup based on which ROI yielded the best within-subject performance.

**Figure 2:**
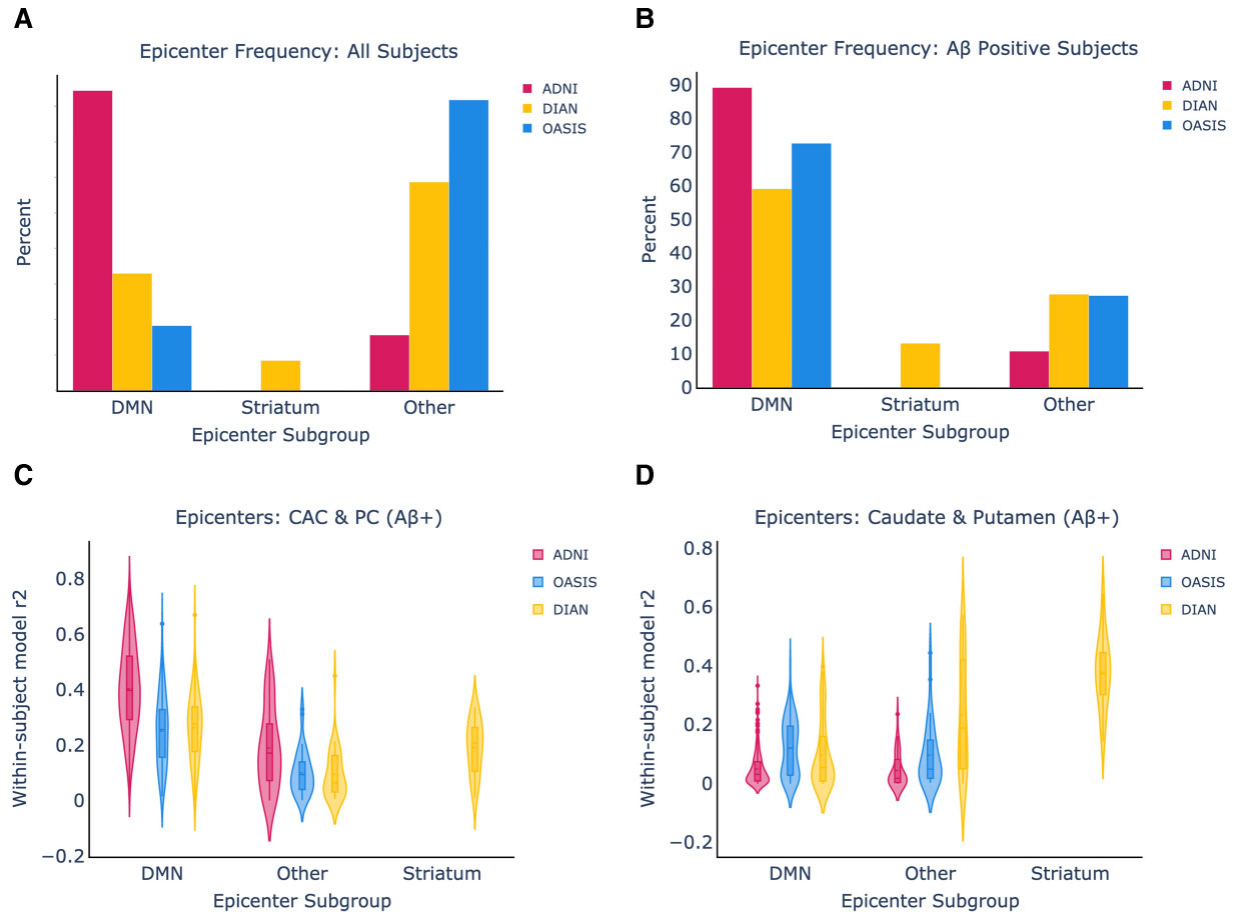
**Epicentre frequency and within-subject performance across all datasets.** (**A**) Epicentre frequency across all subjects in each dataset. (**B**) The same information when only amyloid-beta positive subjects are included from each dataset and epicentre group, using only amyloid-beta positive subjects. (**C**) The ESM within-subject performance is shown using the CAC and posterior cingulate as epicentres. (**D**) The ESM within-subject performance is shown using the caudate and putamen as epicentres.

In Fig. 2A and B, we show the relative breakdown of epicentre subgroups within the datasets in all subjects, and in amyloid-beta positive subjects only. In amyloid-beta positive subjects from ADNI and OASIS, 89.2% of amyloid-beta positive ADNI subjects and 72.7% of amyloid-beta positive OASIS subjects have a DMN epicentre, whilst the remaining subjects fall into the Other category. In the DIAN dataset, there was substantially more heterogeneity, with 59.1% of amyloid-beta subjects falling into the DMN group, 13.1% into the striatum group, and 27.7% into the Other group.

We next assessed the performance of the ESM in each ‘epicentre subgroup’ across different model epicentres. We hypothesized that ESM within-subject fit using the caudate and putamen as epicentres would be highest within the DIAN striatum epicentre subgroup, and this was substantiated by the results (Fig. 2B). Encouragingly, we found that the ESM within-subject fit using the CAC and PC as epicentres was highest in the DMN epicentre subgroups across all the datasets, and it remained high in the Other subgroup for ADNI. The CAC + PC model fit continued to be higher in ADNI than OASIS (KS = 0.42, *P* = 1.6e-12) and DIAN (KS = 0.41, *P* = 7.3e-11) within the DMN groups. Within the DIAN dataset, the striatal model significantly out-performed the CAC + PC model in the striatal epicentre subgroup (KS = 0.67, *P* = 2.15e-4).

### Epicentre subgroup in DIAN associated with distinct whole-brain amyloid-beta patterns and age at symptom onset

Next, we were interested in parsing the heterogeneity observed within the DIAN dataset with respect to best within-subject epicentre. Specifically, we sought to evaluate any differences in whole brain amyloid-beta pattern and demographics.

**Figure 3:**
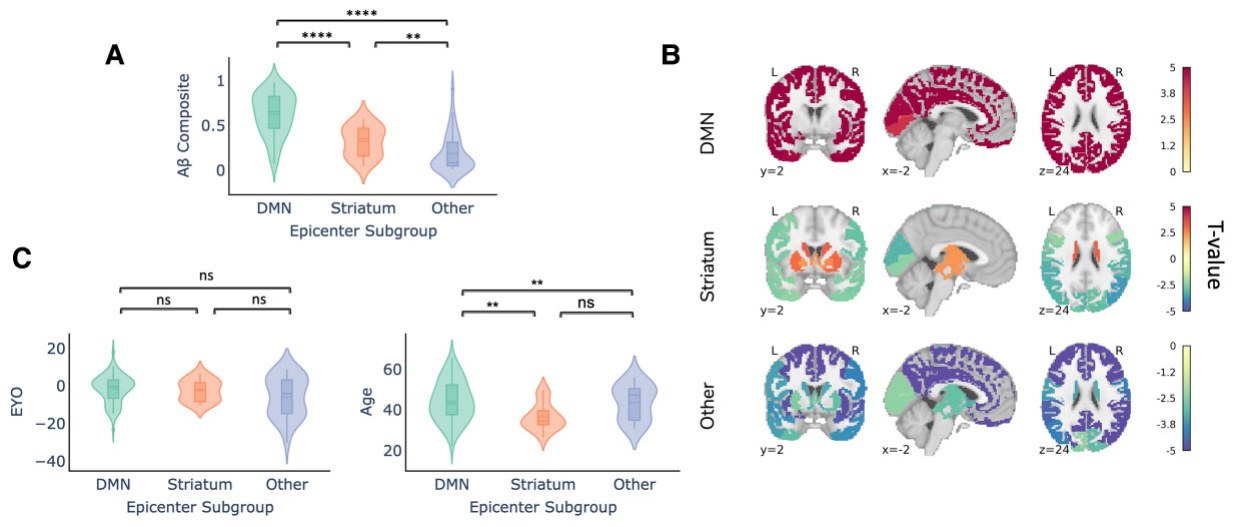
**Demographic differences across epicentre subgroups in DIAN (only amyloid-beta positive individuals).** (**A**) Within-subject amyloid-beta composite signal across the epicentre subgroups. (**B**) Comparison of whole-brain amyloid-beta signal across the epicentre sub-groups. Regions are colour-coded based on their t-value for the particular group, with red indicating that there is more amyloid-beta signal in the respective group compared with the other two groups. (**C**) Within-subject EYO and age differences across the epicentre subgroups were compared using a Mann–Whitney–Wilcoxon test. There were no significant differences for age whereas the DMN group was significantly older than the striatum and other group (DMN versus striatum: *U* = 1091, *P* = 0.003; DMN versus other: *U* = 2089, *P* = 0.005 two-tailed with Bonferoni correction).

As expected, we reaffirmed that individuals in the Other subgroup had significantly lower global cortical amyloid-beta-PET signal (Fig. 3A), suggesting these subjects to be ‘false positives’. In other words, individuals with ‘Other’ (i.e. not DMN or striatal) epicentres tended to be low amyloid amyloid-beta+ individuals, for whom the model was likely fitting non-specific or off-target binding.

We further examined whole-brain amyloid-beta pattern differences amongst the different epicentre groups. Individuals whose whole-brain amyloid-beta patterns are best described using a DMN epicentre have more amyloid-beta in the cortex compared with individuals in the other two groups (FDR < 0.05; Fig. 3B). Conversely, individuals in the Other epicentre subgroup had less amyloid-beta everywhere in the brain. Individuals with striatal epicentres showed greater striatal PiB binding, but reduced binding in occipital and lateral temporoparietal cortex.

The epicentre groups were also associated with differences in age. Specifically, whilst the DMN and striatum group did not differ with respect to EYO, individuals in the striatum group were younger than those in the DMN group (Fig. 3C). This may potentially suggest that the striatal epicentre phenotype is associated with a younger age at symptom onset and/or an altered disease time course. We additionally assessed differences between the epicentre sub-groups in DIAN using a multinomial logistic regression model. The multinomial model replicated our previous finding that age significantly contributed to an individual having a DMN or Striatum epicentre, with age being lower in the Striatum group. The Other epicentre subgroup was also younger and more educated than the DMN group. For the DMN-Striatum and DMN-Other comparisons, we report the z test statistics across the covariates in Supplementary Table 1.

### Epicentre reliability across timepoints

With the availability of longitudinal PiB-PET data for a subset of our dataset, we were able to assess how reliably the ESM selects an individual’s epicentre subgroup when presented with data from subsequent timepoints. As shown in Table 1, 124 of the DIAN mutation carriers had two timepoints available, and 44 had three available.

Subjects with a DMN or Other epicentre at timepoint 1 (T1) almost always stay that way at timepoint 2 (T2), whilst there is more variability amongst subjects with a striatal epicentre at T1. This may perhaps indicate that some individuals with a striatal epicentre at T1 are in a temporally short-lived phase whereby amyloid-beta first begins accumulating in the striatum and subsequently in the DMN. In other words, individuals who are advancing with respect to amyloid-beta accumulation may first either show amyloid-beta in the striatum, the striatum then the DMN, or initially in the DMN.

**Figure 4:**
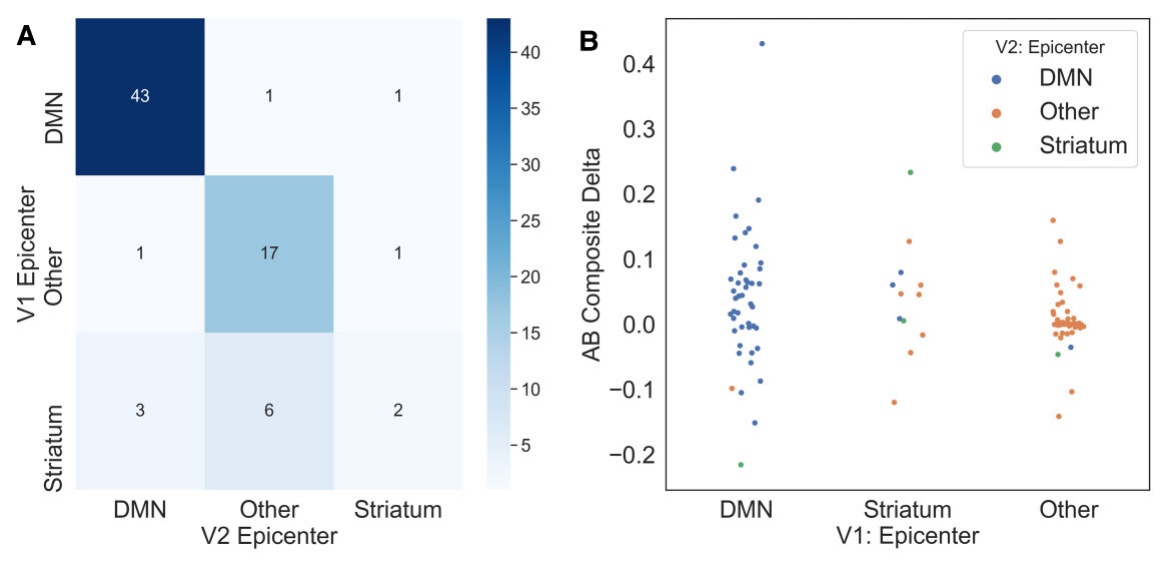
**Evaluating epicentre reliability across timepoints in DIAN.** (**A**) Confusion matrix for epicentre subgroups at timepoint 1 (T1) versus timepoint 2 (T2). Values along the diagonal represent individuals who remain the same epicentre subgroup at visits 1 and 2. (**B**) Swarm plot representing composite amyloid-beta change in each T1/T2 epicentre subgroup combination.

To address this issue of conversion from a striatal epicentre to a different epicentre, we assessed change in composite amyloid-beta deposition probabilities across the different T1–T2 epicentre combinations. We find that individuals who persist with either a striatum or DMN epicentre, or switch from a striatal to DMN epicentre, are gaining amyloid over time (Fig. 4B). We observe that those who switch from a DMN or a striatal epicentre to an ‘Other’ are exhibiting a loss of amyloid-beta signal, possibly due to cortical atrophy.

## Discussion

Throughout this study, we have explored how well a model that simulates the transneuronal spread of amyloid-beta under biologically feasible constraints of amyloid-beta production and clearance can explain regional amyloid-beta probabilities for subjects who are either along the sAD or ADAD continuum. Whilst many cross-sectional studies have attempted to elucidate differences in the regional amyloid-beta patterns across these subtypes of Alzheimer’s disease, the present study provides a direct comparison of hypothetical spreading patterns of amyloid-beta using a mechanistic model.

The ESM generates within subject trajectories of amyloid-beta accumulation, and we leveraged this to assess potential heterogeneity across subjects with respect to the earliest locations of amyloid-beta. Several earlier PiB-PET studies in ADAD have compared which areas begin to accumulate amyloid-beta earliest in the disease time courses of ADAD and LOAD. These studies have reported significantly more amyloid in the striatum in presymptomatic ADAD versus presymptomatic LOAD,^8^ and it has been suggested that different mutation types may contribute to heterogeneity amongst individuals with ADAD.^10,30^ We found that there was a portion of subjects in the DIAN dataset whose regional amyloid-beta patterns were best reproduced using a striatal epicentre. All but two of these subjects were amyloid-beta positive, suggesting that the results were not driven by false positive signal. Furthermore, these subjects could be distinguished from those with a DMN epicentre by their younger age and younger age of symptom onset, lending support to the idea that this group represents an ADAD-specific phenotype distinct from one characterized by initial amyloid-beta spread from ROIs in the DMN. However, one of the difficulties with interpreting this result lies in the small percentage of subjects with a best fitting striatal epicentre. It is difficult to disentangle whether this striatal epicentre group is truly a separate group for whom amyloid-beta definitively begins accumulating solely in the striatum, or the result of these subjects being imaged during a short dynamic time period, or perhaps both. We also found that, in the DIAN dataset, individuals in the ‘Other’ epicentre subgroup were more educated than the DMN subgroup. Given that the ‘Other’ epicentre group had less amyloid, this finding supports previous literature showing a potential protective effect of education on amyloid accumulation in ADAD individuals.^31^

However, not all subjects’ amyloid-beta patterns were best recapitulated using a striatal epicentre, and this was supported by the group-level findings. Whilst a hypothetical striatal epicentre explained more variance in the DIAN dataset than in both the ADNI dataset and our validation dataset, OASIS, a DMN epicentre still explained more variance in DIAN within the entire amyloid-beta positive cohort. This may suggest that the amyloid-beta pattern profiles are not homogeneous amongst ADAD mutation carriers and that there are individuals who are more similar to sAD patients with respect to amyloid-beta. We were able to address this in part by showing that there is a subgroup of DIAN participants whose amyloid-beta patterns are explained as well as the ADNI cohort’s when using the caudal anterior cingulate and PC as epicentres.

Our findings provide data-driven corroboration of a neuropathological study finding that ADAD mutation carriers have increased striatal vulnerability to accumulate amyloid-beta due to the regional distribution and metabolism of APP.^32^ The same study showed an increased accumulation of striatal tau in ADAD mutation carriers compared with sAD individuals, and previous simulations of tau spreading in sAD shed additional light on how amyloid-beta facilitates the spread of tau and influences its spatial localization.^14^ In tandem, a study in ADAD has indicated that striatal amyloid is a better predictor than cortical amyloid of both tauopathy and cognitive decline in ADAD mutation carriers.^33^ With availability of tau-PET data for the DIAN cohort, it would be worthwhile to assess this relationship whilst accounting for the epicentre subgroup differences.

In light of mounting evidence for striatal and network-level involvement in ADAD, both with respect to amyloid-beta and tau, a recent study found that frontostriatal circuits are structurally and functionally impacted by APP and PSEN1 mutations.^34^ Specifically, the APP gene increased functional connectivity and altered axonal integrity in the caudate to rostral middle frontal gyrus tract. Whilst the ESM and other mechanistic spreading models reproduce the spread of amyloid-beta over a static network reflecting anatomical connectivity in health, these results, along with those from a separate study evaluating the sequence of changes in anatomical connectivity in elderly individuals’ brains over the course of sAD progression,^35^ suggest that amyloid-beta affects the circuits or networks via which it spreads.

One objective of this study was to reproduce the findings in^13^ in an independent dataset. One of the issues we observed when modelling group-level results was that of a significant disparity in overall amyloid-beta levels across the three datasets. In particular, the OASIS dataset had a high percentage of younger, cognitively normal adults who were amyloid-beta negative. As we discussed in the Results section, the ESM appears to be sensitive to low levels of amyloid-beta—i.e. the ESM is fit to non-specific or off-target signal not reflecting true pathology, and this would have a particularly large impact on within-subject results for the most likely epicentre(s). As such, we opted to focus on amyloid-beta positive subjects for the within-subject analyses. When we limited our analysis to amyloid-beta subjects, we found that the results across ADNI and OASIS were on par with one another, with a vast majority of subjects being best described by an epicentre that overlaps with the default mode network. This observation is in line with previous data driven approaches used in both cross-sectional and longitudinal studies to discern which regions begin to show increased amyloid-beta in early stage sAD.^35,36^

This study has several limitations that pertain to measurement of amyloid-beta, anatomical connectivity and the ESM methodology. One limitation faced when directly comparing the ADNI and DIAN sets is that the PET data was collected using the AV45 radiotracer in ADNI and the PiB tracer in DIAN/OASIS. Additionally, we sought to use the results in the original ESM publication as a benchmark, and this required using the derivatives that had been produced for that paper. Both OASIS and DIAN had been processed using PUP, and there were subsequently differences in the way that the PET scans were corrected for motion and co-registered to the MRI scans. As had been reported in,^14^ there are many different choices that can be made in a PET data processing pipeline and the connectivity matrix, and the downstream effects include variable model fit. To determine the best epicentre and by extension epicentre subgroup for each subject, we selected the bilateral ROI that yielded the best within-subject fit, but this method ignores potentially close values across ROIs.

Despite these limitations, our study made several important advances. We show that the majority of amyloid-beta positive subjects in three independent datasets had whole-brain amyloid-beta patterns best reproduced using epicentres overlapping with the DMN. The presence of the younger striatal epicentre subgroup in only the DIAN dataset supports the importance of analyzing differences in individual trajectories, as variability in ADAD disease courses may have important implications for efforts to reduce amyloid-beta burden and improve cognitive impairment.

## Supplementary Material

fcac085_Supplementary_Data

## Abbreviations

ADAD =: autosomal-dominant Alzheimer’s disease
ADNI =: Alzheimer’s Disease Neuroimaging Initiative
DIAN =: Dominantly Inherited Alzheimer Network
DMN =: default mode network
ESM =: epidemic spreading model
OASIS =: Open Access Series of Imaging Studies
sAD =: sporadic Alzheimer’s disease
